# Large Iodine Variability in Retail Cows’ Milk in the U.S.: A Follow-Up Study among Different Retail Outlets

**DOI:** 10.3390/nu15143077

**Published:** 2023-07-08

**Authors:** Janet M. Roseland, Katherine M. Phillips, Bryan T. Vinyard, Todor Todorov, Abby G. Ershow, Pamela R. Pehrsson

**Affiliations:** 1 Methods and Application of Food Composition Laboratory, Agricultural Research Service, U.S. Department of Agriculture, Beltsville, MD 20705, USA; janet.roseland@usda.gov; 2 Biochemistry Department, Virginia Tech, Blacksburg, VA 24061, USA; kmpvpi@vt.edu; 3 Statistics Group, Northeast Area, Agricultural Research Service, U.S. Department of Agriculture, Beltsville, MD 20705, USA; bryan.vinyard@usda.gov; 4 Office of Regulatory Science, Center for Food Safety and Applied Nutrition, Food and Drug Administration, U.S. Department of Health and Human Services, College Park, MD 20740, USA; todor.todorov@fda.hhs.gov; 5 Office of Dietary Supplements, National Institutes of Health, Bethesda, MD 20892, USA; abby.ershow@nih.gov

**Keywords:** iodine, milk, dairy, variability, intake, database

## Abstract

In a previous study, large variability in iodine content was found among samples of store brand retail milk at a single time point in a sampling taken from 24 nationwide U.S. locations for the USDA FoodData Central database, but the sampling plan was not designed to detect differences among locations. This follow-up study was carried out to evaluate iodine levels in retail milk across the U.S. over time. Milk samples (2% fat) were collected bimonthly in fourteen locations for one year and analyzed in duplicate. Control materials were used to support accuracy of results and ensure precision across analytical batches. The overall mean and standard error (SE) for iodine concentration were 82.5 (7.0) µg/240 mL serving, which was comparable to the previous national mean [85.0 (5.5) µg/240 mL]. A similar wide range among individual samples was detected (27.9–282 µg/240 mL). For some locations, the mean iodine concentration differed significantly from others, and differed from the national average by amounts ranging from −47 µg to +37 µg per serving. The between-sample range within location was large for some (up to 229 µg/serving) and minimal for others (as little as 13.2 µg/serving). These findings suggest iodine intake from some retail milk supplies could be over- or underestimated relative to the national average, even if the national average is suitable for population-wide intake estimates.

## 1. Introduction

The adequacy of iodine intake is a current worldwide public health concern, especially for women of reproductive age, since iodine is essential for brain and neurological development in fetal and early life [1]. The daily recommended intake (DRI) for iodine is 90 to 130 µg for birth to age 13, 150 µg for ages 14 and older, 220 µg during pregnancy, and 290 µg during lactation [2]. Along with iodized salt, cows’ milk is among the most important food sources of iodine in the U.S. [3,4]. Thus, assessment of dietary adequacy for iodine requires robust data on the typical contribution of cow’s milk and food products made from it.

As part of an effort to develop a comprehensive database of the iodine content of U.S. foods and dietary supplements, we assayed cows’ milk sampled from 24 retail outlets across the U.S. [5], selected according to the USDA’s statistically based plan for the National Food and Nutrient Analysis Program (NFNAP) [6]. Our data including the national mean were first published in the USDA national food composition database in 2021 [7]. The nationwide average (n = 96 samples) was 85.0 µg/240 mL serving, with a standard error of 5.5. The range among individual values was 31–251 µg/240 mL, illustrating high variability. High iodine variability in important food sources of iodine had also been noted by the U.S. Food and Drug Administration’s Total Diet Study [3]. Factors contributing to the content and variability of iodine in milk have been well established [5], so the observed variability was not unexpected. However, sources of variability could not be discerned from the one-time sampling design, which was designed to obtain a nationwide average, so it was not possible to evaluate whether the variability was random or reflected differences among retail supplies. Therefore, we sought to sample more frequently (every other month) to characterize the nature of the variability among various locations across the country over a year’s time.

The key question in the current study was whether iodine in milk consistently differed either among any specific retail sources or between a given regional retail source and the national average. While the first study defined the degree of variability in iodine content that might be experienced by different people residing and buying milk in different locations (i.e., national variation), this study was designed to detect the degree of variability experienced by a single person residing and buying milk in one location over time (i.e., local variation).

## 2. Materials and Methods

### 2.1. Sampling Plan

**Figure 1:**
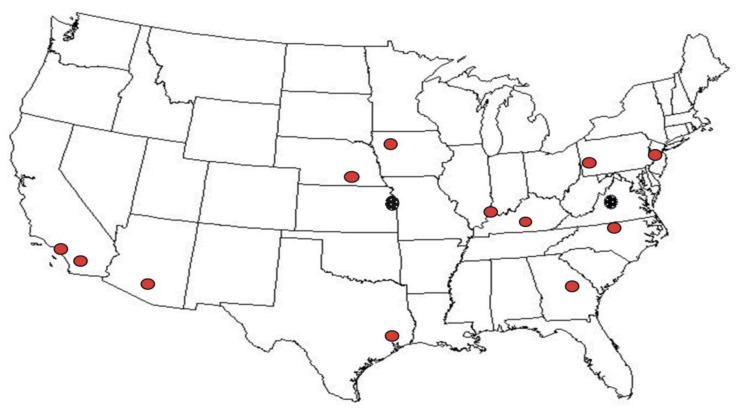
Location of the 12 retail outlets identified using the USDA’s national sampling plan [8] that were re-sampled bimonthly (red dots) and two additional locations sampled monthly (black dots).

We defined a retail supply as a particular brand and milk fat level procured from the same retail outlet, of a brand likely to be consistently chosen by a typical consumer. Twelve retail outlets at locations depicted in Figure 1 were chosen from among the 24 locations in the USDA’s national sampling plan [8] that had been used for the estimation of the nationwide average in our first study [5]. The twelve locations were randomly selected by a statistician to ensure that selection was representative of the U.S. population and food supply.

One half-gallon (1.89 L) carton of 2% fat milk was collected from each outlet every other month from May 2021 to March 2022 (n = 6 per location; total 72 samples). Additionally, samples were obtained monthly for a year at each of two retail outlets located near the two research sites (Blacksburg, VA and Shawnee, KS between December 2020 and November 2021 to estimate within-location variability with greater accuracy (n = 12 per location; total 24 samples). The brand occupying the largest display space (typically the store brand or major local/regional brand) at each outlet was sampled [5] and was consistent within each outlet. Samples were shipped to the Food Analysis Laboratory Control Center (Virginia Tech, Blacksburg, VA, USA), where subsamples were taken and stored at −60 °C, as previously described [9].

### 2.2. Iodine Analysis

Each milk sample was analyzed in duplicate for iodine by inductively coupled plasma mass spectrometry (ICP-MS) after extraction of the sample with potassium hydroxide and stabilization with ammonium hydroxide and sodium thiosulfate (AOAC 2012.15 [10]) or tetramethyl ammonium hydroxide (FDA Elemental Analysis Method 4.13 [11]). The samples were batched with a 2% milk control material (“2% Milk CC”), and the samples from each outlet were distributed across multiple assay batches (~15 samples per batch) so that estimates of day-to-day analytical variability would not be confounded by variability among sampling locations. NIST SRM^®^ 1869 Adult/Infant Nutritional Formula II, having a certified iodine concentration (National Institute of Standards and Technology, Gaithersburg, MD, USA), was analyzed in duplicate in two analytical batches. The HorRat ratio (“HorRat”) was calculated for the assayed concentration (µg/100 g) in sample and control replicates, as RSD_assayed_/((Mean_assayed_/100/1,000,000)^^−^.^1505^), where RSD is the percent relative standard deviation and was considered acceptable if ≤2.0, according to Horwitz and Albert [12]. Results for the 2% Milk CC were expected to be within the mean ± 2SD of all of our previous analyses (27.8–32.5 µg/100 g). If a control sample value was outside the acceptable range, the batch of samples would be re-analyzed. No batches required reanalysis.

### 2.3. Data Analysis

For nutritional relevance, analytical concentrations expressed in µg/100 g were converted into units of µg/240 mL, corresponding to a 1 cup (8 fluid oz.) serving size for fluid milk [13]. We established the density of milk as 243.7 g/240 mL. All statistical analyses were performed with SAS^®^ software (v9.4 TS1M7, SAS Institute; Cary, NC, USA, 2021) using the means of sample duplicates, for a total of 96 data points in the final data set.

Iodine values were log-transformed due to their positive skew and general increase in variance with increase in mean. The statistical analyses were conducted on log values, and estimates obtained from the SAS PROC MIXED ANOVA models were then back-transformed from log to the original (non-log) scale. However, estimates (and standard errors) of differences between location and nationwide means were necessarily obtained using the original (non-log) scale to maintain interpretability.

A one-way ANOVA model with a fixed location effect (more specifically, the heterogeneous variance group “hvg” model described below) was used to obtain location least squares means, 95% confidence intervals of means, and 95% prediction intervals for individual samples; conduct pairwise location means comparisons using Sidak *p*-value adjustment, with experiment-wise Type I error rate <5%; and specify contrasts to test for differences between each location mean and the nationwide mean. The model was initially specified to obtain a unique estimate of within-location variance for each location to examine within-location heterogeneity across locations. This initial saturated model was reduced by assigning each location to a group, determined by the similarity (within four times magnitude) of its within-location variance with that of other locations in that group. A non-significant likelihood ratio test (LRT) [14] indicated that the reduced model (with four different magnitudes of within-location variance) fit the data as well as the saturated model (with a unique within-location variance estimate for each location). The degrees of freedom saved by using this hvg model (instead of estimating a unique variance for each location) increased the model’s error degrees of freedom and, hence, increased the power of all obtained statistical tests. The nationwide average 95% confidence interval used a mean squared error calculated as an average of the 4 hvg variances, each weighted by the number of locations in the associated variance group.

## 3. Results and Discussion

### 3.1. Sample Descriptive Information

The samples from 14 different retail stores across the U.S. included 13 different brands. The processor for each carton of milk was identified by its code on the package labels [15], indicating that all samples from each given retail location came from the same processor. For each location, the processor was situated in the same state as the retail store, or else in an adjoining state. Among the 12 retail stores in the bi-monthly sampling, the processor was the same as in our first study except for two outlets, so most locations had supplier continuity.

### 3.2. Quality Control

The mean analyzed iodine concentration for the total of 15 samples of the 2% Milk CC was 30.3 µg/100 g (range 28.5–32.7 µg/100 g) with a HorRat of 0.2, and all values were within the mean ± 2SD of 17 previous values, including the three samples analyzed in the first study [5]. For the total of 96 samples analyzed in replicate, the mean and median difference between replicates were 0.9 and 1.1 µg/240 mL, respectively (range 0.0–9.0 µg/240 mL). The two results for NIST SRM^®^ 1869 Adult/Infant Nutritional Formula II reference material (124 and 129 µg/100 g) were within the certified concentration range of 113–143 µg/100 g [16]. These quality control data support the accuracy of the results for the milk samples, excellent measurement precision across analytical batches, and minimal analytical uncertainty in the iodine concentration reported for each sample.

### 3.3. Milk Iodine Content

**Figure 2:**
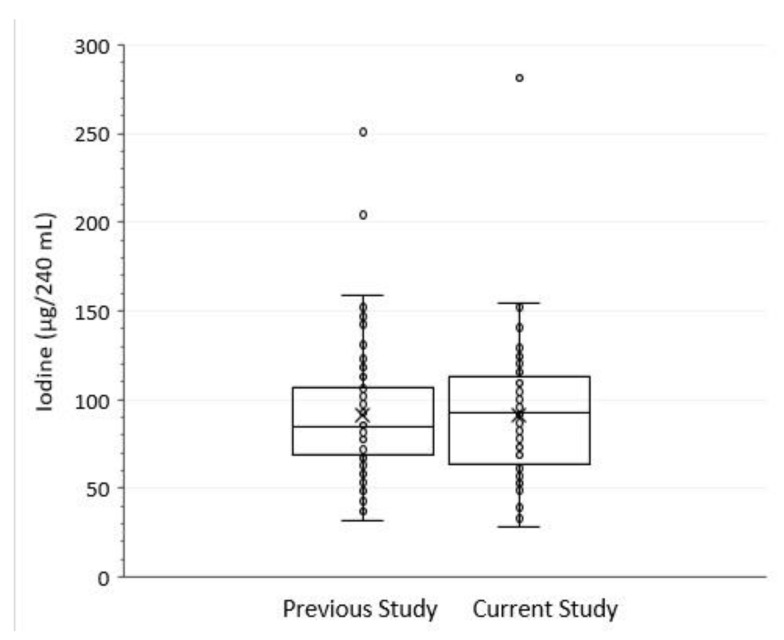
Iodine concentration (µg per 240 mL serving) in the previous study [5] and current study. Data points represent iodine concentration in individual samples. The vertical bars are 95% confidence intervals for the individual sample values. (×) indicates the observed mean. The lower, middle, and upper horizontal lines in the boxes respectively indicate the first quartile, median, and third quartile.

The overall mean iodine concentration of 82.5 µg/240 mL serving with a 95% confidence interval (n = 96 samples) of 67.1–101.4 did not differ from the previous study [5] (mean = 85 and 95% confidence interval of 74–97 µg/240 mL, respectively) (Figure 2). This result supports the predictive population-wide estimate of the average amount of iodine contributed by retail milk in the U.S. as reported in the USDA’s FoodData Central Foundation Foods database [7].

**Figure 3:**
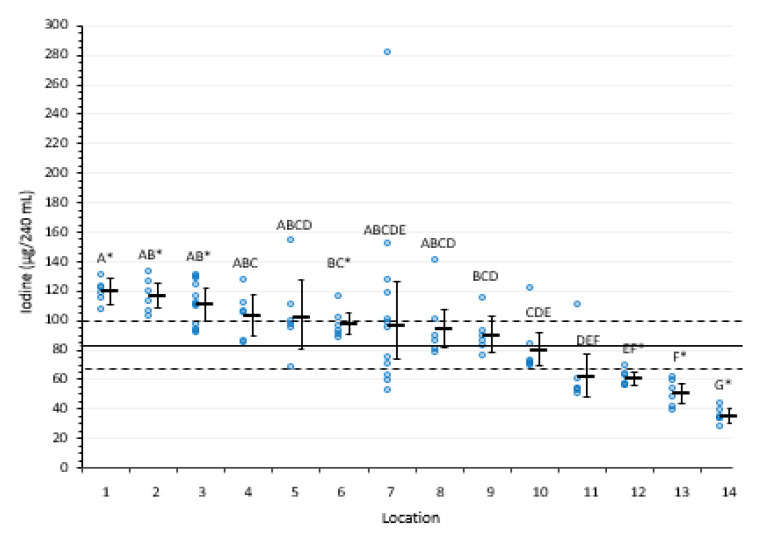
Iodine content of individual milk samples (○) and the mean ± 95% confidence interval for each location for 14 retail outlets in the U.S. Different capital letters indicate a statistically significant difference between means (*p* < 0.001), and * denotes a location mean that differs from the overall average of 82.5 µg/240 mL serving (solid line, with a 95% confidence interval of 67.1–101.4 µg/240 mL (dashed lines)).

Figure 3 shows the iodine content by retail location. Locations 12, 13, and 14 had mean iodine contents of 60.6, 50.1, and 35.3 µg/240 mL, respectively, that were significantly lower relative to other locations and the national average of 82.5 µg/240 mL. Location 1 had mean iodine content of 113 µg/240 mL, significantly higher compared to all other locations and to the national average.

Because duplicate samples had no more than 4.5% RSD for any sample (median 0.5%), the between-sample variance within location is notable in some cases. Variance was lowest and did not differ among locations 1, 12, 13, and 14, which had within-location ranges (maximum minus minimum) of 13.2 to 23.5 µg/240 mL. On the other end of the spectrum, variance was largest within outlets 5, 7, 8, and 11, which had within-location ranges (maximum minus minimum sample concentration) of 52.2 to 229 µg/240 mL. Location 7 had one sample with an iodine content of 281 µg/240 mL, which is 129 µg/240 mL more than any other sample from that location, but the range without this value was still large (99.4). The remaining locations had similar variance, intermediate between lowest and highest groups.

Sources of iodine in milk and factors affecting its concentration and variability have been extensively examined in controlled studies [17,18,19,20,21,22] and mainly result from feed supplementation and use of iodine in sanitation practices [5]. The National Research Council of the National Academies has established a maximum allowable amount of supplemental iodine for U.S. dairy cows [23,24], and the federal Pasteurized Milk Ordinance (PMO) [15] includes extensive recommendations for providing a safe milk supply throughout the production chain, including permitted concentrations for iodine used for sanitation [25]. Existing regulations cite only the upper allowable limits, so significant variation in practice is possible within these limits. Each state has an agency legislating milk safety and quality [26], but we could not find any state regulations differing from the federal guidelines. Although this study provides expanded data regarding variability among sampling locations, it was not designed to determine specific causes of differences among locations.

Although regulations generally limit excessive iodine in milk, there is evidence that some regulations might encourage practices to severely limit iodine in milk production. It is worth noting that Bruhn et al. [27,28] reported on trends and voluntary changes in iodine use in dairy production in California in the 1980s, intended to lower the iodine content of milk, and in our study both CA locations sampled (Locations 13 and 14; Figure 3 and Table 1) had iodine concentrations notably lower than the national average and most other locations. Guidelines within a group of regional dairy farms or particular processors could in turn affect the contribution of iodine in a particular milk supply.

In total, milk from seven locations had a mean iodine concentration per 240 mL serving that differed (*p* < 0.0001) by 10% or more of the DRI from the nationwide mean of 82.5 µg/serving (Table 1), ranging from −47 to +37 µg/240 mL (−31% to +25 of the DRI). Importantly, these amounts based on consuming 240 mL per day would increase in proportion to intake. For example, daily consumption of 720 mL (3 cups), which might be typical of a child or teenager and is also the recommended intake for pregnant women [29,30], would provide −141 to +111 µg iodine (−94% to +74% of the DRI) relative to 255 µg (the estimated iodine amount in 3 cups based on the national mean). Additionally, the study confirmed that random variability within a retail supply can be substantial even if the location average does not differ from the national average and also confirmed that variability within retail supplies differs.

## 4. Conclusions

The magnitude of the difference in iodine concentration of many individual samples relative to the mean was nutritionally significant. Whereas a one-cup (240 mL) serving of milk would provide 67% of the iodine DRI for adults based on the national mean, the contribution from a one-cup serving at the observed minimum (31.4 µg) or maximum (251 µg) would provide 21% or 167% of the DRI, respectively. For an intake of 3 cups (720 mL) per day, which might be typical of many children and adolescents, this range becomes more dramatic. Thus, understanding the contribution of milk, as well as other food sources of iodine, is an important aspect of understanding population intake patterns and developing dietary guidance for individuals and at-risk groups.

These findings suggest caution in using the national U.S. average of 85 µg/240 mL serving, as published in the USDA database [7], for anything other than nationwide studies characterizing population average iodine intake, or for developing guidance on dietary sources of iodine for various dietary patterns. Applying the reported national average milk iodine concentration has the potential for large over- or underestimation of iodine intake from milk consumed from a particular retail supply, such as for a single-location clinical trial or evaluation of iodine intake in an individual or a localized population. For research diets or controlled feeding studies, an accurate estimate of iodine intake would require analyzing samples of any milk provided to participants. For most applications, such as characterizing the distributions of population or group iodine intakes or developing guidance on dietary sources of iodine as appropriate for various dietary patterns, the national U.S. average of 85 µg/240 mL serving, as published in the USDA database [7], is a reliable value.

## 5. Future Directions

This work highlights the need for food composition databases to include different summary statistics besides means (e.g., number of analyses, standard deviation, median, distribution of nutrient concentrations for each food). This is especially important for nutrients such as iodine with highly variable concentrations in commonly consumed food sources. The data from this study can improve estimates, particularly when assessing individuals with intakes at the tail ends of the distribution (i.e., percentage that may be deficient and those with excessive usual iodine intakes). Carriquiry et al. emphasized the importance of data for variability of iodine and recommendations for assessing population intake of iodine [31]. Work is underway for developing estimates of total usual iodine intakes of the U.S. population and population sub-groups. Data from the National Health and Nutrition Examination Study (NHANES) are being linked to iodine concentration values from the USDA, FDA, and ODS-NIH Database for the Iodine Content of Common Foods [32]. More importantly, the data from this study will also be used to estimate the percentage of the population and population subgroups not meeting the estimated average requirement or those exceeding the tolerable upper intake level.

## Figures and Tables

**Table 1:** Sample locations with mean iodine content (µg/240 mL) differing (*p* < 0.0001) from the overall mean of 82.5 (with 95% confidence interval: 67.1–101.4).

Location	Location Mean	Difference between Location Mean and Overall Mean	Standard Error of Difference
1	119	36.9	3.4
2	117	34.2	5.6
3	110	28.0	4.2
6	97.4	14.9	5.6
12	60.6	−22.0	3.4
13	50.1	−32.0	3.4
14	35.3	−47.0	3.4

## Data Availability

Data obtained in this study are presented in Table 1 and Figure 2 and Figure 3.
